# Supplementary material for: Transgenic Overexpression of HDAC9 Promotes Adipocyte Hypertrophy, Insulin Resistance and Hepatic Steatosis in Aging Mice
Source: Biomolecules. 2024 Apr 18;14(4):494. doi: 10.3390/biom14040494 (PMC11048560; doi:10.3390/biom14040494)
Supplement: Supplementary file 1 [file biomolecules-14-00494-s001.zip › biomolecules-2894379-supplementary.pptx]

## Slide 1
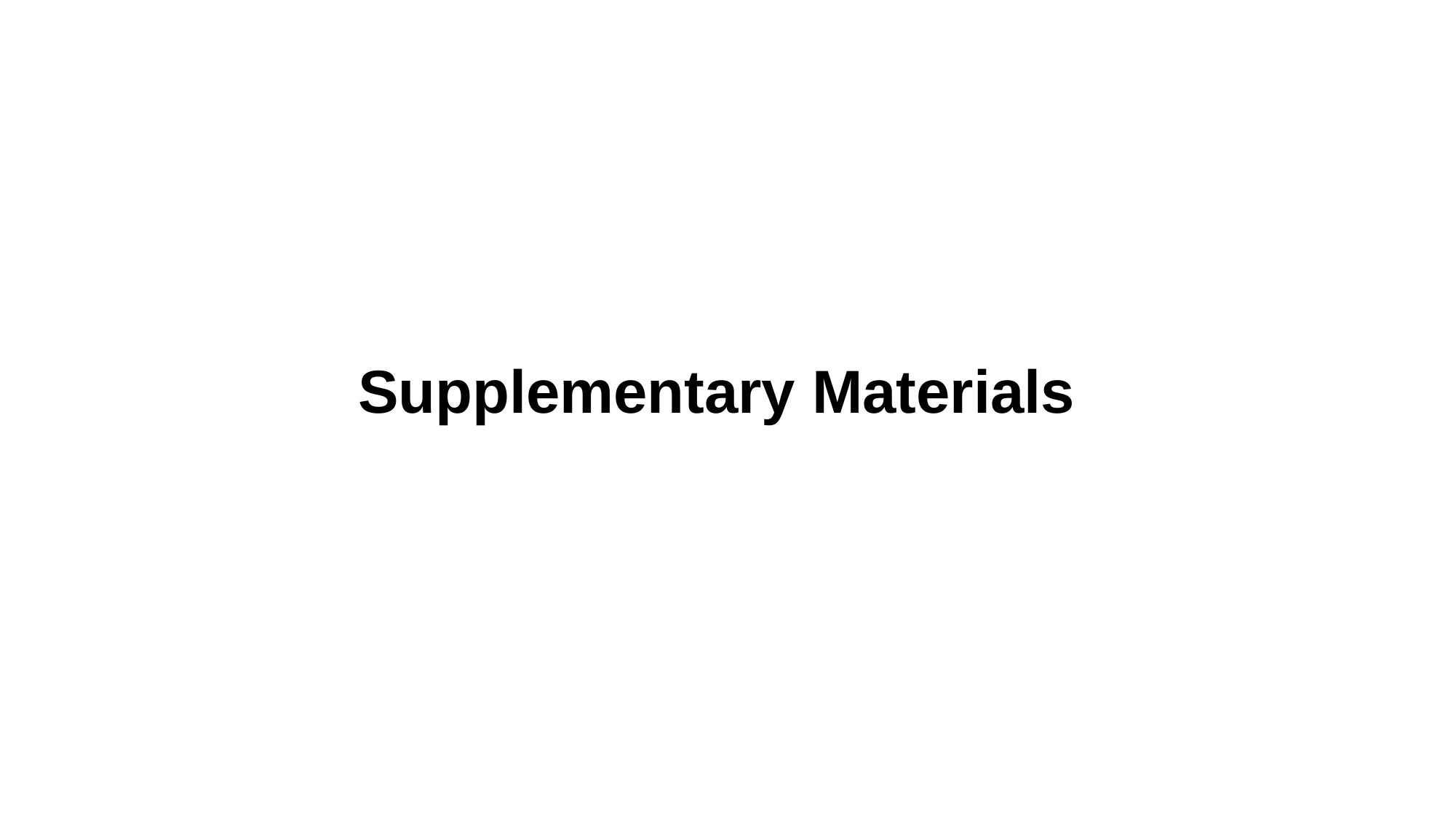

# Supplementary Materials

## Slide 2
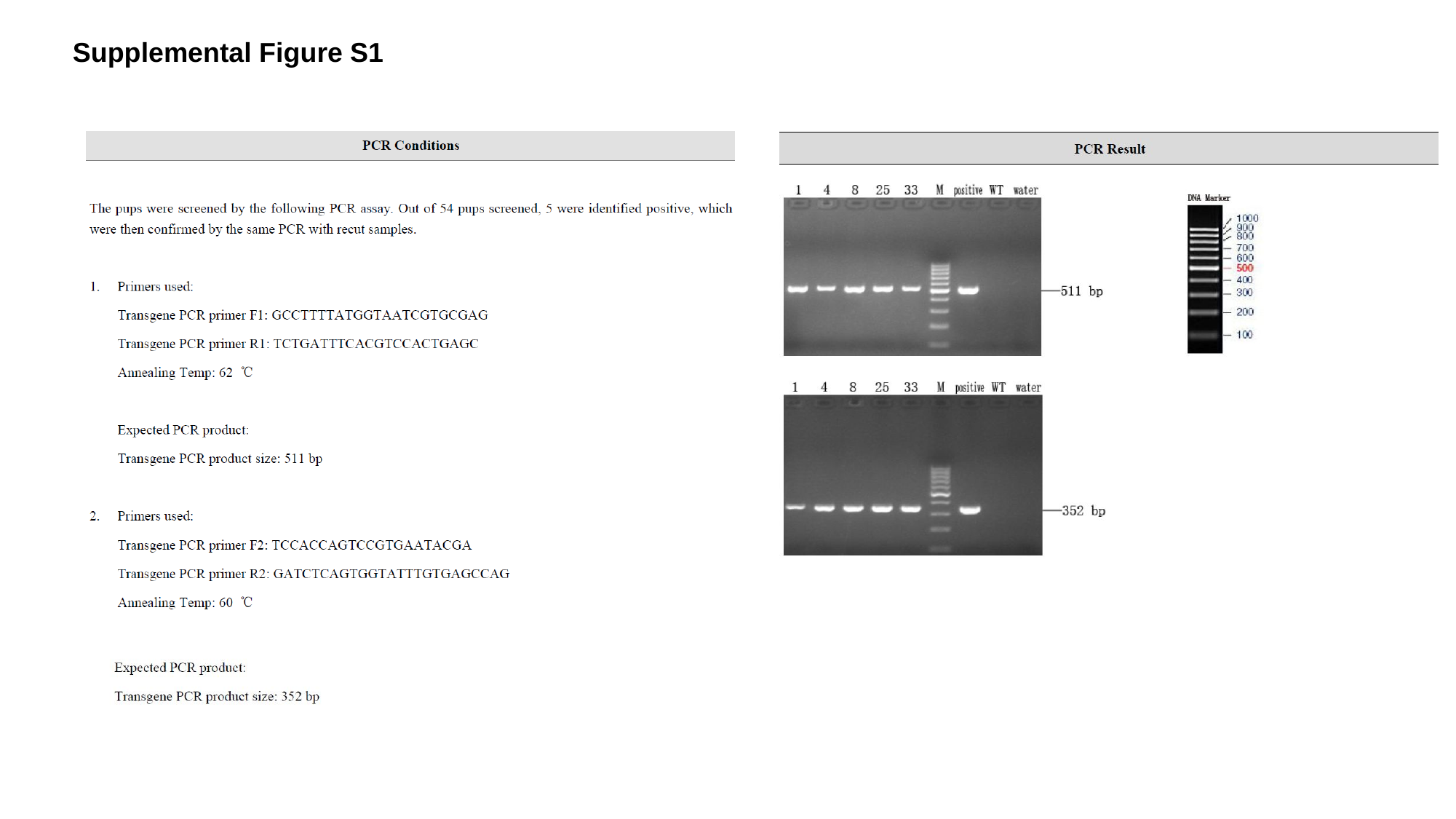

Supplemental Figure S1

## Slide 3
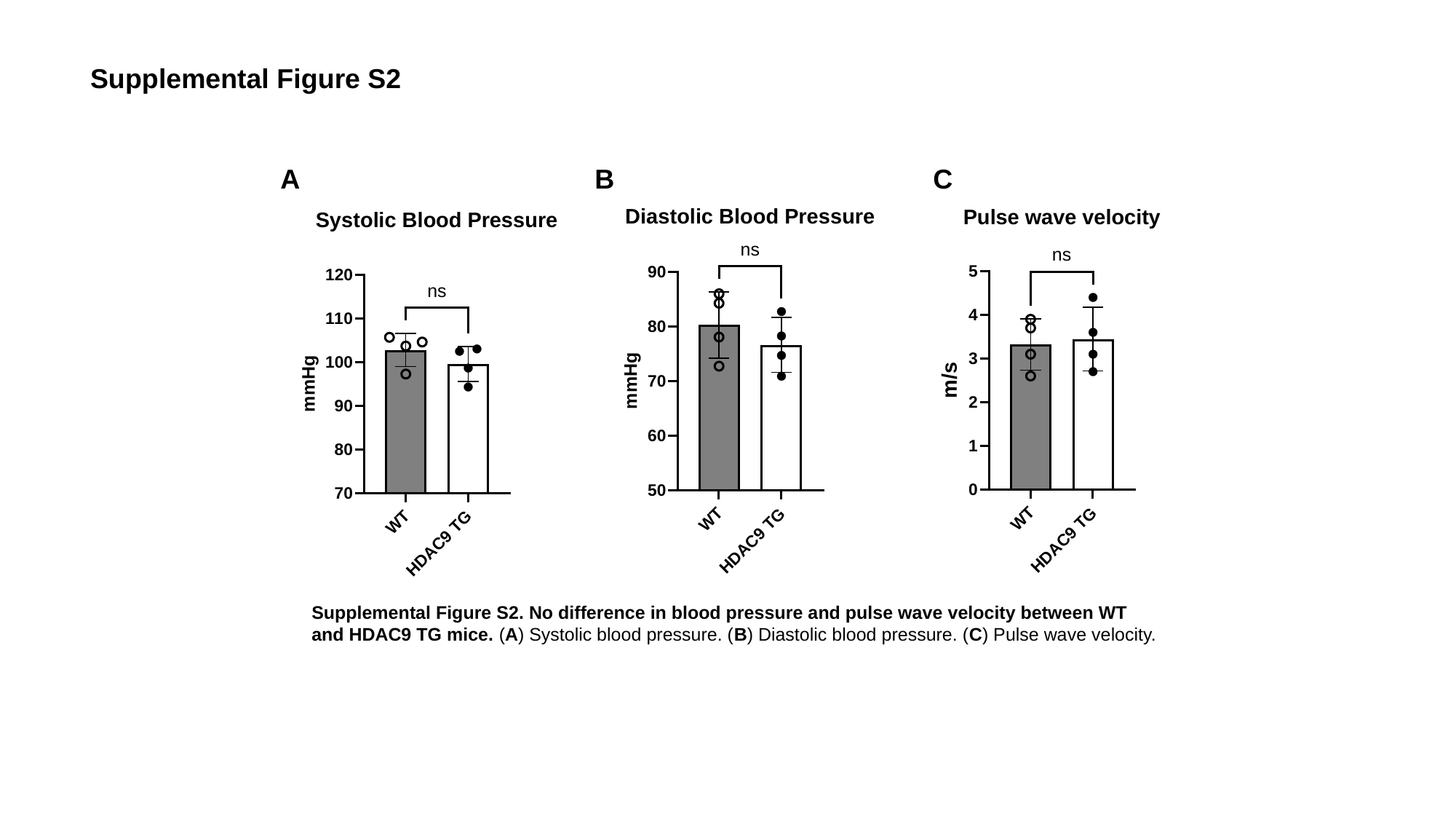

Supplemental Figure S2
A
B
C
Supplemental Figure S2. No difference in blood pressure and pulse wave velocity between WT and HDAC9 TG mice. (A) Systolic blood pressure. (B) Diastolic blood pressure. (C) Pulse wave velocity.

## Slide 4
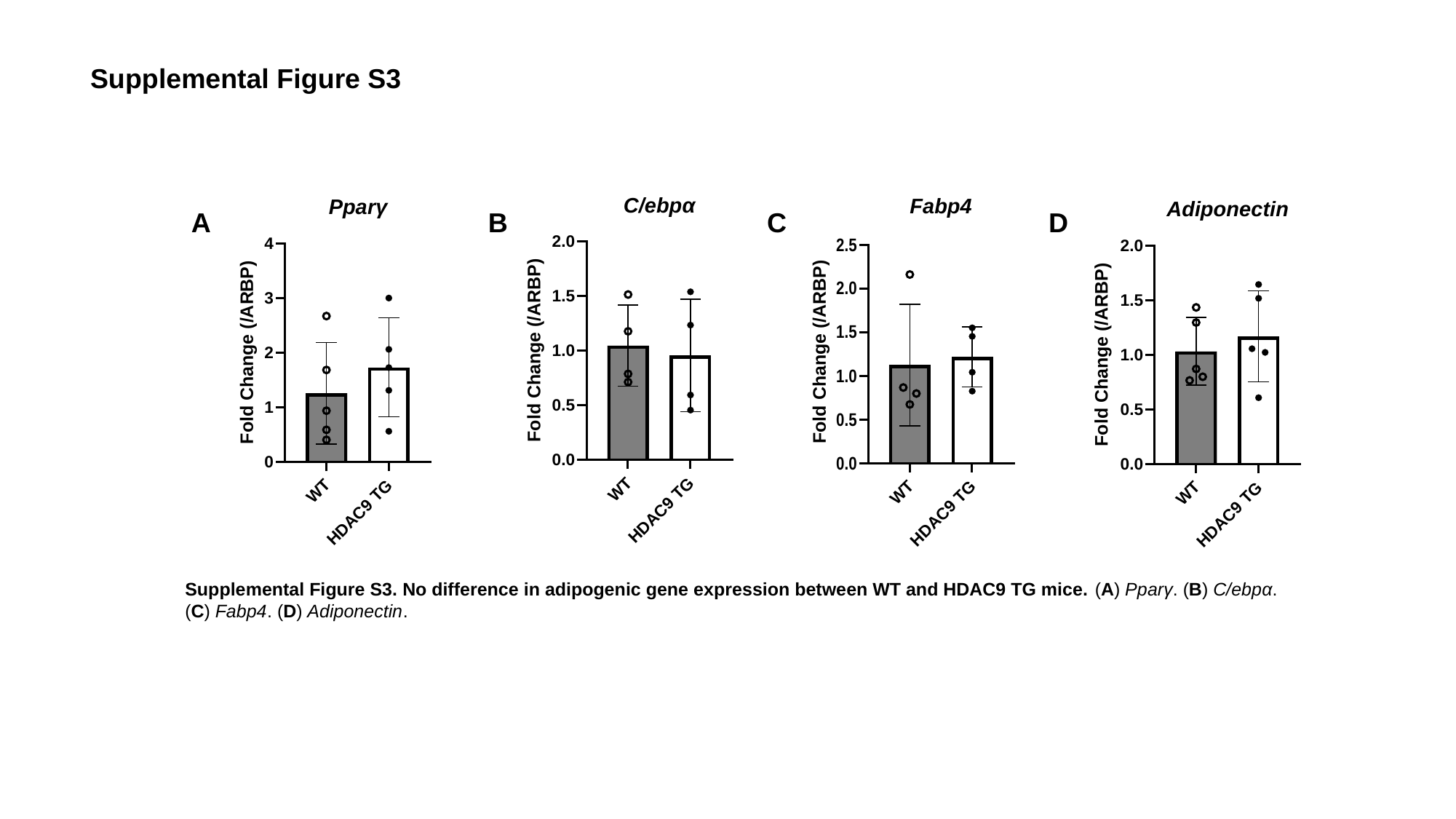

Supplemental Figure S3
A
B
C
D
Supplemental Figure S3. No difference in adipogenic gene expression between WT and HDAC9 TG mice. (A) Pparγ. (B) C/ebpα. (C) Fabp4. (D) Adiponectin.

## Slide 5
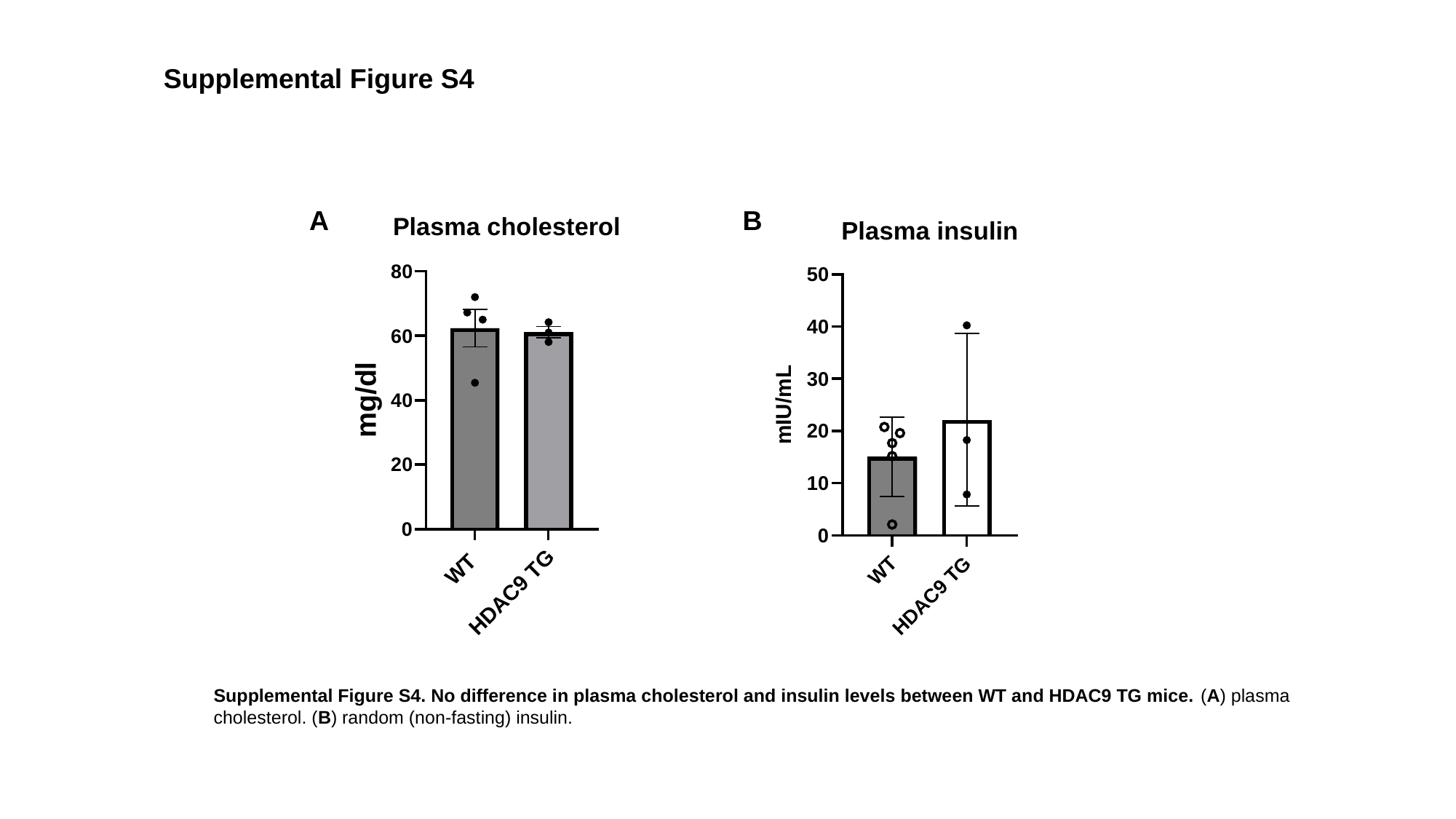

Supplemental Figure S4
A
B
Supplemental Figure S4. No difference in plasma cholesterol and insulin levels between WT and HDAC9 TG mice. (A) plasma cholesterol. (B) random (non-fasting) insulin.

## Slide 6
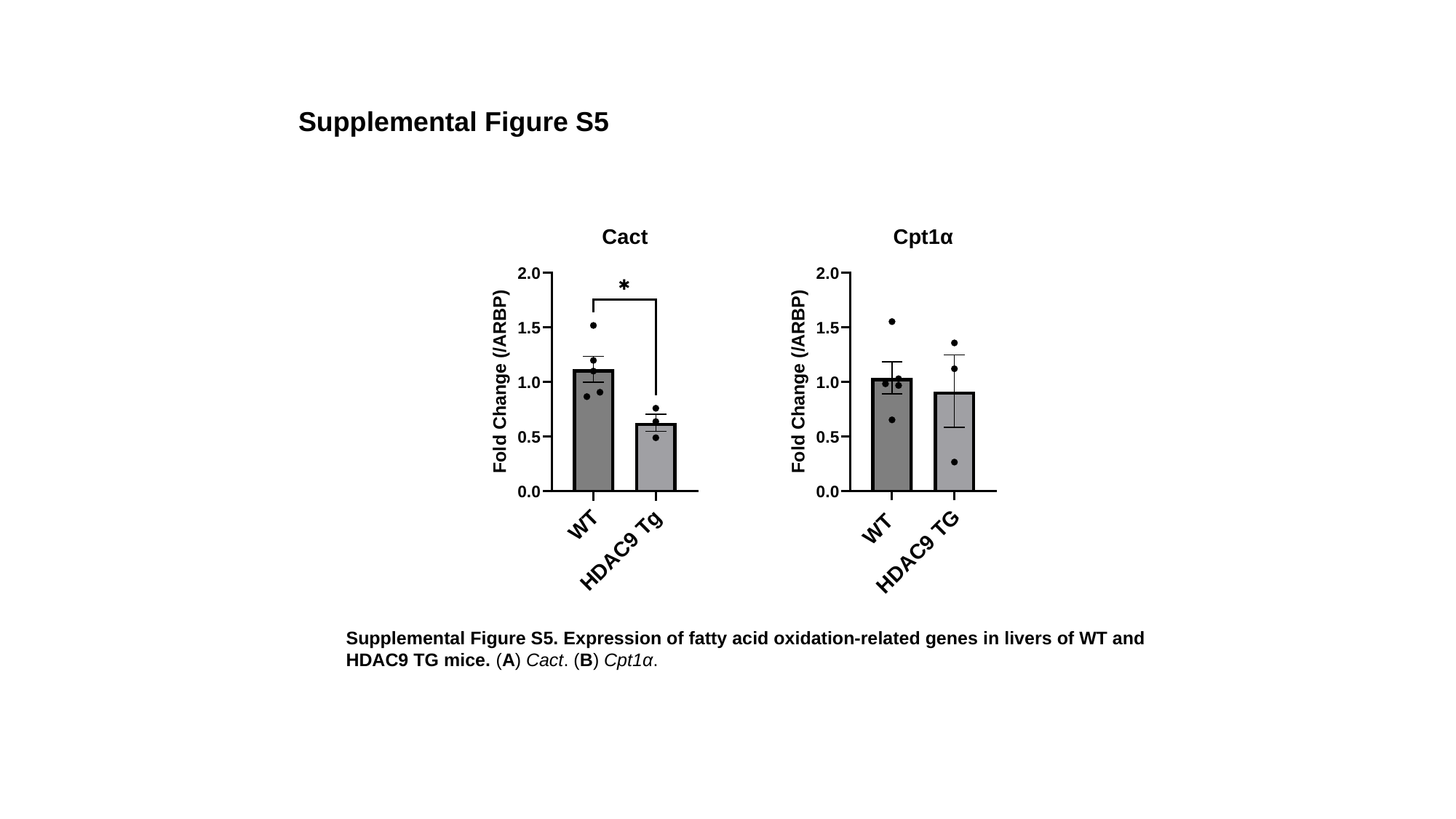

Supplemental Figure S5
Supplemental Figure S5. Expression of fatty acid oxidation-related genes in livers of WT and HDAC9 TG mice. (A) Cact. (B) Cpt1α.

## Slide 7
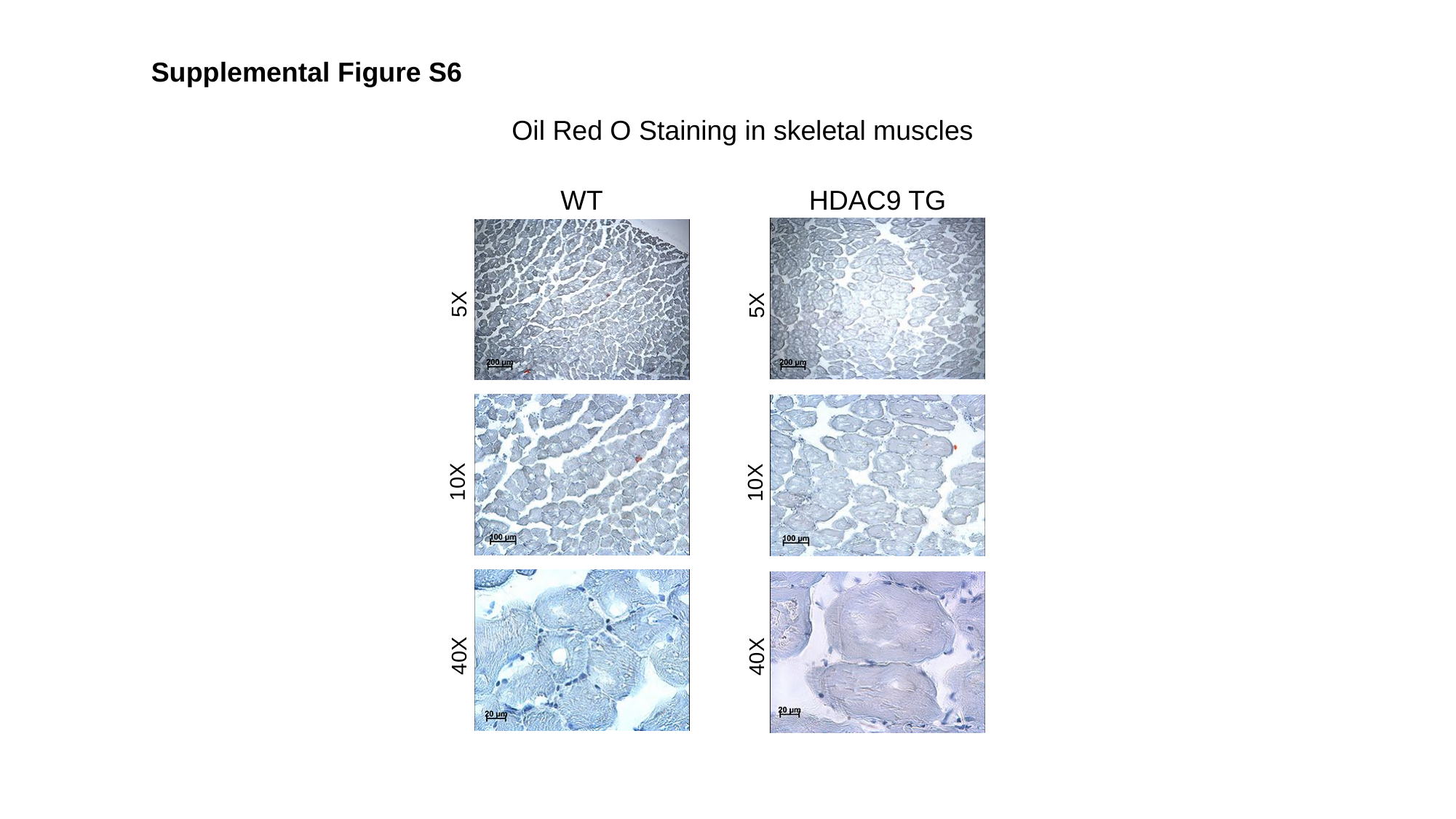

Supplemental Figure S6
Oil Red O Staining in skeletal muscles
WT
HDAC9 TG
5X
5X
10X
10X
40X
40X

## Slide 8
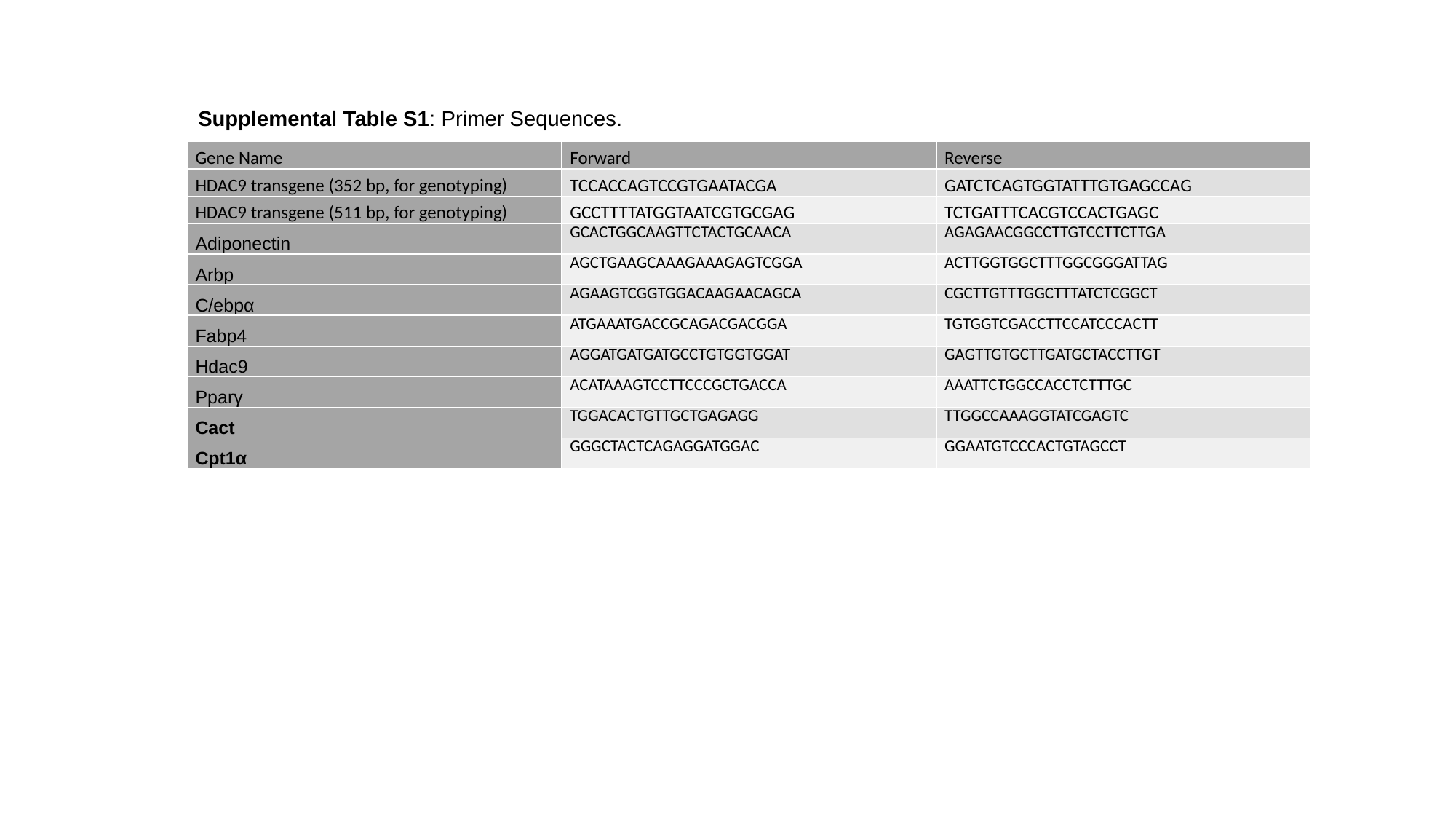

Supplemental Table S1: Primer Sequences.
| Gene Name | Forward | Reverse |
| --- | --- | --- |
| HDAC9 transgene (352 bp, for genotyping) | TCCACCAGTCCGTGAATACGA | GATCTCAGTGGTATTTGTGAGCCAG |
| HDAC9 transgene (511 bp, for genotyping) | GCCTTTTATGGTAATCGTGCGAG | TCTGATTTCACGTCCACTGAGC |
| Adiponectin | GCACTGGCAAGTTCTACTGCAACA | AGAGAACGGCCTTGTCCTTCTTGA |
| Arbp | AGCTGAAGCAAAGAAAGAGTCGGA | ACTTGGTGGCTTTGGCGGGATTAG |
| C/ebpα | AGAAGTCGGTGGACAAGAACAGCA | CGCTTGTTTGGCTTTATCTCGGCT |
| Fabp4 | ATGAAATGACCGCAGACGACGGA | TGTGGTCGACCTTCCATCCCACTT |
| Hdac9 | AGGATGATGATGCCTGTGGTGGAT | GAGTTGTGCTTGATGCTACCTTGT |
| Pparγ | ACATAAAGTCCTTCCCGCTGACCA | AAATTCTGGCCACCTCTTTGC |
| Cact | TGGACACTGTTGCTGAGAGG | TTGGCCAAAGGTATCGAGTC |
| Cpt1α | GGGCTACTCAGAGGATGGAC | GGAATGTCCCACTGTAGCCT |

## Slide 9
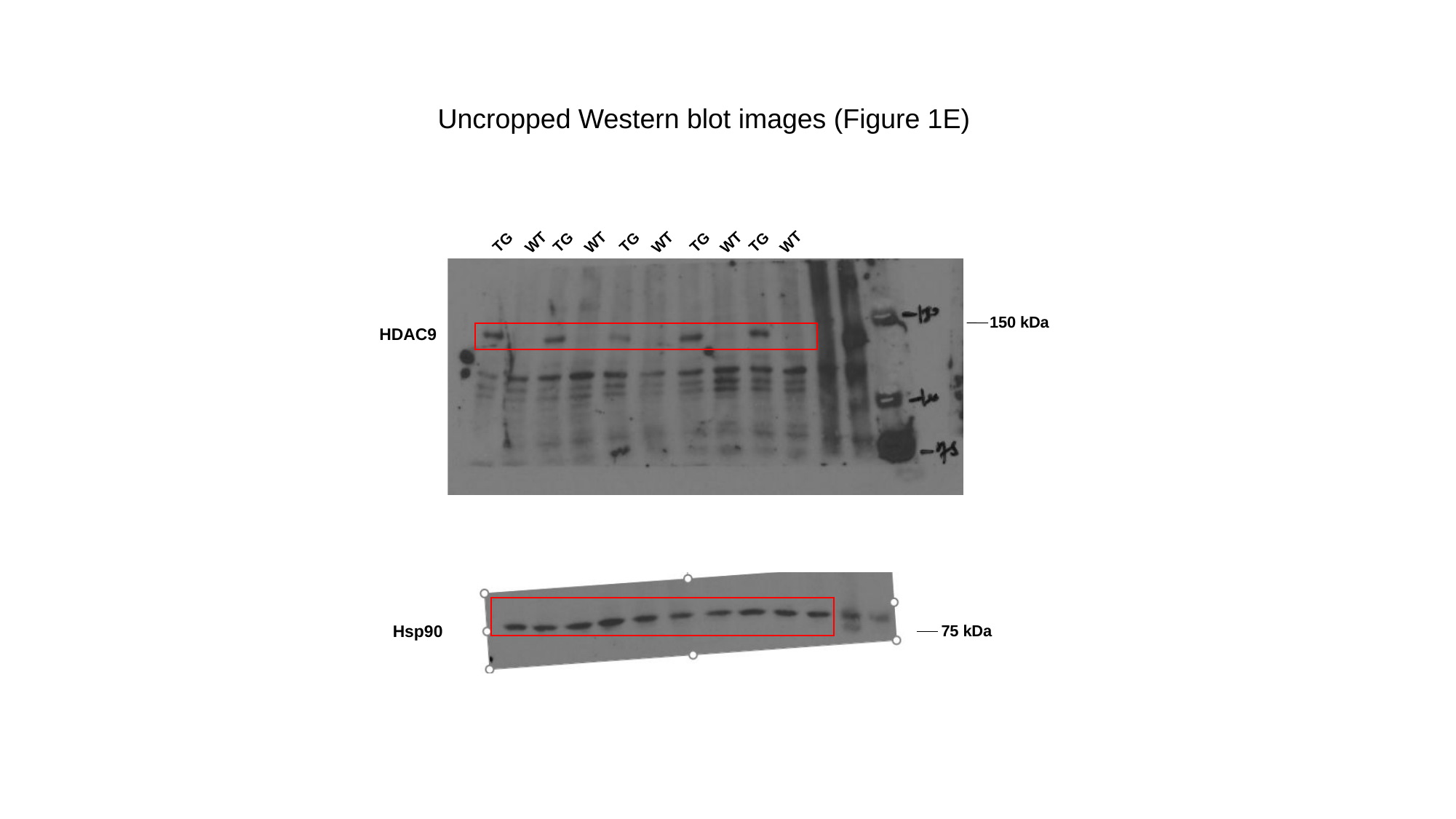

Uncropped Western blot images (Figure 1E)
TG
WT
WT
TG
TG
WT
TG
WT
TG
WT
150 kDa
HDAC9
Hsp90
75 kDa
