# Supplementary figures and images for: Transgenic Overexpression of HDAC9 Promotes Adipocyte Hypertrophy, Insulin Resistance and Hepatic Steatosis in Aging Mice
Source: Biomolecules. 2024 Apr 18;14(4):494. doi: 10.3390/biom14040494 (PMC11048560; doi:10.3390/biom14040494)

Original images for blots/gels

Figure 1B

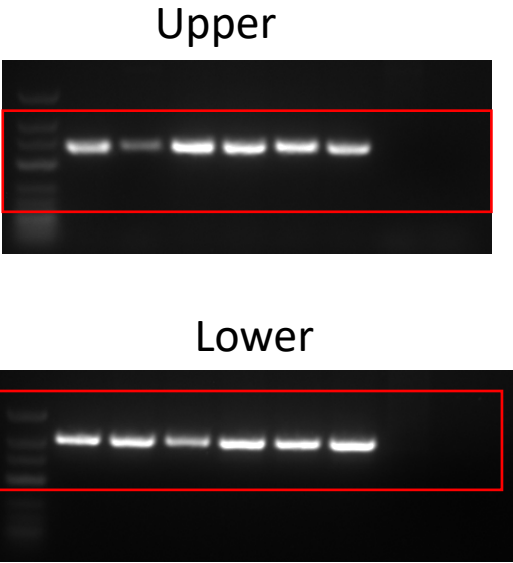

Figure 1E

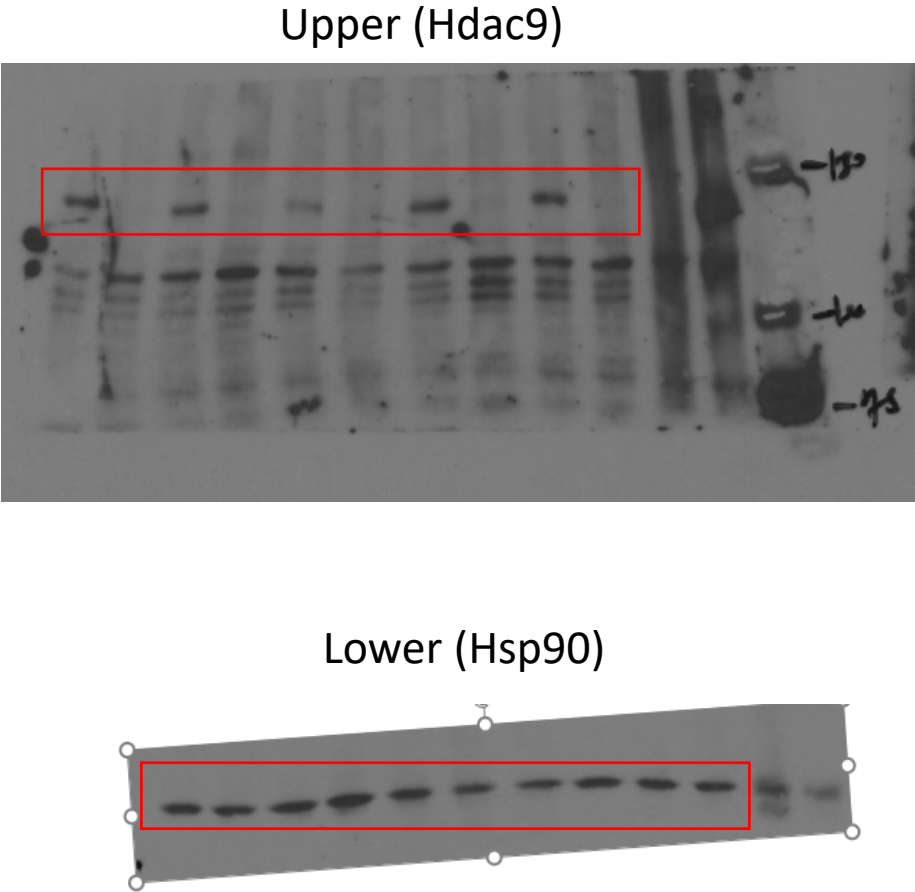

Supplement: Supplementary file 1 [file biomolecules-14-00494-s001.zip › biomolecules-2894379-original-images.pdf]
